# Iridoids derived from Valeriana jatamansi Jones alleviates neuroinflammation and blood spinal cord barrier permeability after spinal cord injury by activating the Nrf2/HO-1 signaling pathway

**DOI:** 10.3389/fphar.2025.1597719

**Published:** 2025-07-18

**Authors:** Yongzhi He, Jiachun Lu, Rizhao Pang, Lijuan Ding, Yunyun Wang, Hua Xiao, Chao Cheng, Yushan Luo, Xiaoming Hu, Wenchun Wang

**Affiliations:** ^1^ Department of Rehabilitation Medicine, Xichong County People’s Hospital, Nanchong, Sichuan, China; ^2^ Department of Rehabilitation Medicine, General Hospital of the Western Theater Command, Chengdu, Sichuan, China; ^3^ Sichuan Provincial Clinical Medical Research Center for Traditional Chinese Medicine Orthopedics and Sports Rehabilitation, Chengdu, Sichuan, China; ^4^ Chengdu Eighth People’s Hospital (Geriatric Hospital of Chengdu Medical College), Chengdu, Sichuan, China; ^5^ College of Medicine, Southwest Jiaotong University, Chengdu, Sichuan, China; ^6^ The Second Affiliated Hospital of Guangzhou University of Chinese Medicine (Guangdong Provincial Hospital of Chinese Medicine), Guangzhou, Guangdong, China

**Keywords:** spinal cord injury, neuroinflammation, Nrf2/HO-1 signaling pathway, blood-spinal cord barrier, Valeriana jatamansi Jones

## Abstract

**Background:**

Valeriana jatamansi Jones, a globally utilized medicinal plant, exhibits favorable pharmacological effects against depression and tumors. Iridoids derived from V. jatamansi (IRFV) promote recovery following spinal cord injury (SCI). Inflammation and disruption of the blood-spinal cord barrier (BSCB) represent key pathological processes in SCI. However, the specific effects of IRFV on neuroinflammation and BSCB integrity remain unexplored.

**Methods and Results:**

This study aims to elucidate the functional significance and molecular mechanisms by which IRFV modulates neuroinflammation and preserves BSCB function following SCI. Experimental results demonstrated that IRFV treatment significantly enhanced locomotor recovery in SCI models. Moreover, IRFV reduced macrophage infiltration and inhibited inflammatory mediator secretion, effectively attenuating the neuroinflammatory response. IRFV also mitigated BSCB permeability alterations by suppressing tight junction disruption and structural damage. *In vitro* experiments revealed that IRFV attenuated oxygen-glucose deprivation/reperfusion (OGD/R)-induced endothelial cell damage and tight junction protein degradation, suggesting a potential mechanism for its BSCB protection. Critically, the protective effects of IRFV were abolished upon suppression of the Nrf2/HO-1 pathway, demonstrating its essential role in this process.

**Conclusion:**

In conclusion, our study demonstrates that IRFV treatment activates the Nrf2/HO-1 signaling pathway, thereby suppressing neuroinflammation, mitigating blood-spinal cord barrier damage, and promoting recovery from SCI, thus highlighting its therapeutic potential.

## 1 Introduction

Spinal cord injury (SCI) is a major health concern (Crispo et al., 2023), characterized by the loss of motor and sensory function below the injury level resulting from damage to the spinal cord caused by external forces or other factors (Anjum et al., 2020). Statistics indicate an SCI incidence rate of approximately 569.7 cases per million people annually, with approximately 66,374 new cases occurring each year (Jiang et al., 2021). This imposes a substantial mental and economic burden on patients and their families. Despite significant advancements in SCI treatment modalities such as stem cell transplantation and the development of biomaterials in recent years, clinical efficacy remains suboptimal. Consequently, SCI treatment continues to pose a significant challenge in global medicine (Hu et al., 2023).

The pathological process following spinal cord trauma comprises two distinct phases: initial primary mechanical injury and subsequent progressive secondary injury. While primary injury is irreversible, current SCI treatment strategies primarily focus on mitigating secondary injury (Sterner and Sterner, 2022). The blood-spinal cord barrier (BSCB) serves as a selective interface separating the circulatory system from neural tissue, maintaining spinal cord homeostasis through strict regulation of molecular trafficking and preventing the infiltration of harmful substances, cellular components, and pathogens into the central nervous system (Jin et al., 2021). However, SCI disrupts the BSCB’s tight junction structure and increases its permeability. This disruption triggers the exudation of blood components and facilitates immune cell infiltration into the cord parenchyma, thereby promoting the release of inflammatory mediators and exacerbating post-traumatic neuroinflammation (Deng et al., 2022; Hellenbrand et al., 2021). Research evidence indicates that preserving BSCB integrity—through protecting tight junction structures and modulating vascular permeability—mitigates neuroinflammation and promotes functional recovery after SCI (Tail et al., 2024; Nie et al., 2024). Consequently, reducing BSCB disruption interrupts the secondary injury cascade, establishing it as a critical therapeutic target for SCI treatment.

Valeriana jatamansi Jones is a perennial herb of the family Valerianaceae. Its roots and rhizomes are extensively used in traditional medicine across China, India, and certain American countries (Yang et al., 2021). Traditional applications recognize its pharmacological potential, demonstrating therapeutic effects including sedation, antidepressant activity, analgesia, and antidiarrheal properties. Growing evidence supports its neuroactive potential, with compounds like IRFV and bioactive extracts showing promise in treating neurological disorders (Li et al., 2020). Notably, Lv et al. (2025) demonstrated that Valeriana jatamansi extract ameliorates depressive-like behaviors in mice by modulating vitamin B12-associated ileal microbiota homeostasis. Complementary studies show that ethanol extracts of Valeriana jatamansi attenuate post-traumatic stress responses through dual modulation of cerebral neurotransmitters and hypothalamic-pituitary-adrenal (HPA) axis activity (Yang et al., 2021). Moreover, Dong et al. (2018) identified its rhizome extract as a potent N-type calcium channel inhibitor in oocytes, suggesting promise as a novel therapeutic source for neuropathic pain. Collectively, these findings underscore V. jatamansi’s multifaceted neuropharmacological profile, bridging traditional applications with mechanistic insights and highlighting its translational relevance.

Iridoids derived from Valeriana jatamansi Jones (IRFV), bioactive compounds derived from this plant, exhibit neurorestorative properties following SCI (Wang Y. et al., 2023). Our prior research demonstrated that IRFV administration significantly improved functional recovery in rodent SCI models. This effect is potentially mediated through the upregulation of neurotrophic factors, such as brain-derived neurotrophic factor and nerve growth factor, contributing to neuroprotection. However, the precise molecular mechanisms remain incompletely understood (Xiong et al., 2022). Crucially, studies indicate IRFV can mitigate structural damage to the BSCB (Zhang et al., 2021). Given the functional and protective similarities between the blood-brain barrier (BBB) and BSCB in maintaining neural microenvironment stability (both critical for nervous system homeostasis) (Uchida et al., 2019), the potential of IRFV to attenuate BSCB disruption and thereby promote neurological repair after SCI warrants further investigation.

The current investigation was designed to systematically examine IRFV-mediated modulation of BSCB integrity and neuroinflammatory responses post-spinal cord trauma using complementary *in vivo* and *in vitro* experimental approaches. Our findings indicate that IRFV treatment facilitates functional recovery and attenuates tissue damage in SCI models, concomitant with reduced immune cell migration and inflammatory mediator secretion, suggesting a potential mechanism involving preservation of BSCB integrity. *In vitro* experiments further demonstrated that IRFV protects the BSCB by alleviating endothelial cell damage and tight junction disruption induced by OGD/R stimulation, potentially through regulation of the Nrf2/HO-1 molecular cascade. This study highlights the therapeutic efficacy and mechanistic pathways of IRFV intervention in SCI, emphasizing its potential clinical benefits.

## 2 Materials and methods

### 2.1 Animal and spinal cord injury models

Seventy-two male SPF SD rats, with a weight of 220–250 g, were purchased from Beijing Sbeifu Biological Co., LTD (Animal license number: SCXK (Beijing) 2023-0010). The animals were housed in a clean and well - ventilated environment. The temperature was kept within the range of 22–26°C, the environment was controlled at 60% relative humidity under a 12-h light-dark cycle. All animal studies were performed in accordance with ethical regulations. The Ethics Committee of the General Hospital within the Western Theater Command provided its approval (Ethics number: 2023EC5-ky040).

To induce anesthesia, 5% sodium pentobarbital was injected intraperitoneally at a rate of 30 mg/kg. Once the animals were fully anesthetized, skin preparation was performed, followed by removal of the T10 lamina. The corresponding segment of the spinal cord was clamped for 10 s using a 110 g medical aneurysm clip (Kjell and Olson, 2016). The wound was then disinfected and sutured. Postoperative care included intramuscular injection of penicillin at a dose of 200,000 U/day for three consecutive days. Manual compression of the bladder was done three times daily to help with urination until the bladder regained its normal function. In the sham operation group, only T10 laminectomy was conducted, without any clamping of the spinal cord.

A total of 72 rats were randomly allocated into three groups: sham, SCI, and IRFV, with 24 rats in each group. Rats within the IRFV group underwent intragastric administration of IRFV solution at a dose of 10 mg/kg daily. This dose had been determined as the most suitable in previous research. Meanwhile, rats in the sham and SCI groups received an equal-volume intragastric administration of the vehicle on a daily basis for 28 days. In each group of 24 rats, eight were randomly selected for 28-day behavioral testing, while the remaining 16 were euthanized after 7 days of intervention for subsequent analyses, including ELISA, Western blot, immunofluorescence, and other assays.

### 2.2 Extract the Iridoid-rich fraction of Valeriana jatamansi Jones

Valeriana jatamansi Jones (VJJ was obtained from Hebei Renxin Pharmaceutical Co., Ltd. The extraction process went like this: VJJ was ground into powder form. Subsequently, the crude VJJ powder was subjected to extraction using 70% ethanol. The extraction ratio at the beginning was 8:1, and the extraction time was 24 h, followed by a subsequent extraction at a ratio of 6:1 for an additional 12 h. The extracts that were obtained were blended together and concentrated under reduced pressure so as to get rid of the smell of residual alcohol, giving rise to an ethanol extract. The ethanol extract was evenly dispersed in water through ultrasonic treatment and then loaded onto a D101 microporous resin column. The D101 microporous resin column boasted an adsorption capacity of 72 mg/g. The adsorption process was carried out at a flow - rate of 1 bed volume (BV) per hour, while the elution process occurred at a flow - rate of 2 BV per hour. The elution procedure consisted of sequential treatments. Initially, 6 BV of water was used to elute the column. Subsequently, 4 BV of 60% ethanol was employed for further elution. At last, 4 BV of 95% ethanol was used for the final treatment. The fraction obtained from the elution with 95% ethanol was collected and concentrated under reduced pressure until it reached a paste-like consistency before being vacuum dried at a temperature of 40°C. The total iridoids in the extract were quantified using ultraviolet spectrophotometry, revealing that the content of iridoids amounted to be approximately 83.25%.

The IRFV solution was prepared using 0.5% sodium carboxymethylcellulose (CMC-Na) as the solvent. IRFV solution was prepared at a concentration of 10 mg/kg (Xiong et al., 2022). One gram of the IRFV extract was weighed and dissolved in 0.5% CMC-Na solution with ultrasound assistance, then diluted to a constant volume of 1 L and stored in the dark at 4°C.

### 2.3 Behavioral tests

Eight rats from each group were selected for behavioral analysis. Behavioral tests were performed before injury and 1, 7, 14, 21, and 28 days after modeling, and the classic BBB score (Basso et al., 1995) was used to evaluate the hindlimb motor function of rats. The specific procedure: the hindlimb movement process of rats was observed for 5 min in the open field and scored. The scores were obtained from two raters who did not know the grouping and scored independently after they were familiar with the scoring criteria. Test scores range from 0 (complete paralysis of the hind limbs) to 21 (gait coordination with normal activity).

### 2.4 Hematoxylin-eosin staining

Spinal cord tissues were placed in 4% paraformaldehyde for fixation for 24 h. After that, they were processed with gradient dehydration and embedded in paraffin to produce paraffin specimens regarding spinal cord injury. Spinal cord tissue sections with a thickness of 4 μm were prepared, commencing from the center of the spinal cord injury. H&E staining was performed to assess the degree of histopathological damage. The procedure included deparaffiinization in xylene solution. After that, it was rehydrated using a graded ethanol series (100%, 90%, 80%, and 70%). Nuclei were stained with hematoxylin dye, and tissues were subjected to treatment with Acid Alcohol Fast Differentiation Solution and Dako Bluing Buffer. Finally, the cytoplasm was stained with eosin dye. After the sections had dried, they were covered and placed under a microscope for observation and analysis.

### 2.5 ELISA test

Fresh spinal cord tissue was collected, and samples were prepared through tissue homogenization. According to the manufacturer’s directions, the levels of IL - 1β, TNFα, IL - 6, and IL-10 in the spinal cord tissue were measured by using an ELISA kit (Elabscience, China). The absorbance at 450 nm was measured by means of a microplate reader.

### 2.6 Evans blue staining

Evans blue cannot cross the BSCB to reach spinal cord tissue under normal physiological conditions. After spinal cord injury, the BSCB is destroyed and loses its role in limiting macromolecular substances. The inability to block macromolecules, including Evans blue, would allow detection of Evans blue in spinal cord tissue. Therefore, the changes of BSCB permeability can be evaluated by detecting the spinal cord tissue of Evan blue.

At post-SCI day 7, a 2% Evans blue solution (2 mL/kg) was introduced via tail vein. Following 2 h of systemic circulation, the injured spinal cord region was collected and homogenized in 50% trichloroacetic acid. The homogenate was centrifuged at 10,000 × g for 10 min, and the supernatant was subsequently gathered. The absorbance of each sample was determined using a microplate reader. The level of Evans blue present in the spinal cord tissue was calculated in accordance with the previously established Evans blue concentration-absorbance standard curve.

### 2.7 Transmission electron microscope examination

The tissues with rich blood vessels in the spinal cord tissue were selected for sampling, and the spinal cord tissue was fixed in 2.5% glutaraldehyde, dehydrated, and embedded. Semi-thin sections were prepared to locate the location of the BSCB, and ultrathin sections were prepared according to the location. BSCB ultrastructure was photographed and analyzed by transmission electron microscopy (JEOL, Japan), and tight junction length and basement membrane thickness were quantified by ImageJ.

### 2.8 TUNEL staining

TUNEL staining was carried out following the protocol provided by the manufacturer. Tissue sections were initially dewaxed using xylene, followed by hydration through a graded ethanol series and a final rinse. Excess water around the tissue sections was removed using filter paper. After that, each sample had 100 μL of the 1× Proteinase K working solution added to it and was then incubated at 37°C for a duration of 20 min. Afterward, to equilibrate the samples, they were incubated in TdT Buffer at 37°C in a humidified chamber for 10–30 min. Next, 50 μL of the labeling working solution was applied to the samples, which were then incubated at 37°C in the dark for 60 min. Then, DAPI working solution was added. The surplus fluid was carefully blotted away with blotting paper, and the slides were covered with a mounting medium having an anti-fluorescence quenching agent. Fluorescence images were captured by a Nikon ECLIPSE CI-L microscope, and the fluorescence intensity was quantified with ImageJ software.

### 2.9 Western blot analysis

Fresh spinal cord tissue and cell samples were obtained. Proteins were extracted in accordance with the instructions of the protein extraction kit (Salarbio, Beijing, China). The extracted proteins were mixed with RIPA buffer and inhibitors and incubated on ice for 30 min. Subsequently, the mixture was homogenized using a tissue homogenizer and centrifuged at 12,000 × g for 10 min at 4°C.

In the electrophoresis step, 40 μg of The protein sample was combined with 5× loading buffer and heated for 5 min to induce protein denaturation. The denatured proteins were separated by gel electrophoresis, transferred to a PVDF membrane, and blocked with 5% skim milk at room temperature for 1 h. The membrane was incubated at 4°C for 24 h with the primary antibodies: ZO-1 (1:3000, 30487-1-AP, Proteintech, Wuhan, China), Occludin (1:3000, 27260-1-AP, Proteintech, Wuhan, China), Nrf2 (1:1000, 16396-1-AP, Proteintech, Wuhan, China), HO-1 (1:1000, 66743-1-IG, Proteintech, Wuhan, China), and β-actin (1:5000, Proteintech, Wuhan, China).

The membrane was washed with TBST for 5 min per wash, followed by incubation with the secondary antibody at room temperature for 1 h. It was then washed three more times with TBST for 5 min each. Protein bands were visualized using an ECL chemiluminescence reagent (Themo Fisher Scientific, USA). The bands were exposed and detected via the ChemiDoc XRS system (Bio-Rad, USA). The experiment was conducted in triplicate. Band gray values were quantified using ImageJ software, and protein expression levels were determined by calculating the ratio of these gray values.

### 2.10 IF staining

Spinal cord tissues were fixed with 4% paraformaldehyde for 24 h. The tissues then underwent gradient dehydration and paraffin embedding. After preparing 4-μm-thick sections, they were deparaffinized and rehydrated through a graded ethanol series. Antigen retrieval was performed in a microwave oven using EDTA antigen retrieval solution (Salarbio, Beijing, China). For cell samples, fixation was carried out with 4% paraformaldehyde for 30 min, followed by two washes with PBS. To block non-specific binding, tissue sections were treated with 5% goat serum and incubated at room temperature for 1 h. Subsequently, primary antibodies including CD68 (1:100, AB283654, Abcam, USA), CD31 (1:100, AB182981, Abcam, USA), and ZO-1 (1:200, 66452-1-Ig, Proteintech, Wuhan, China) were applied, and the sections were incubated overnight at 4°C. After a 30-min room-temperature equilibration, secondary antibodies were added and incubated for 1 h at the same temperature. Following five PBS washes, nuclei were stained with DAPI. Finally, slides were mounted and imaged using a Nikon ECLIPSE CI-L microscope, with fluorescence intensity analyzed via ImageJ software.

### 2.11 Cell culturing and the OGD/R model setup

Human brain microvascular endothelial cells (hCMEC/D3) were purchased from Procell (Wuhan, China). Upon revival, cells were cultured in Dulbecco’s Modified Eagle’s Medium (DMEM, Procell, China) supplemented with 10% fetal bovine serum (FBS, Procell, China) and 1% streptomycin/penicillin (Procell, China). Cultures were maintained in a humidified incubator at 37°C with 5% CO_2_, and the medium was refreshed every 2–3 days. Experiments were initiated when cells reached 80%–90% confluence.

An *in vitro* spinal cord injury model was established via oxygen-glucose deprivation/reoxygenation (OGD/R). The culture medium was replaced with glucose-free, serum-free DMEM, and cells were incubated in a tri-gas incubator (37°C, 1% O_2_, 5% CO_2_, 94% N_2_) for 6 h. Subsequently, cells were reverted to standard DMEM and cultured in a 37°C humidified incubator with 5% CO_2_ for 24 h. At 6 h post-OGD/R, treatment was applied by adding 5 μg/mL Valeriana jatamansi iridoid (prepared in DMEM). The Nrf2 inhibitor ML385 (KKL MED, USA) was dissolved in DMEM at 5 μM and used as needed.

### 2.12 Cell counting kit-8 assay

CCK-8 assays were carried out on the cells at the third passage. A cell density of 2 × 10^4^ cells per well was used for plating in 96-well plates. According to the manufacturer’s directions, CCK-8 reagent was added to each well, and then the plates were placed in a humidified incubator at 37°C for 1 h. The cell viability was assessed by measuring the absorbance at 450 nm with a microplate reader.

### 2.13 *In vitro* endothelial permeability assay

Permeability assays were performed on cells at passage three. The cells were seeded into Transwell chambers at a density of 2 × 10^4^ cells per well (Corning, USA) and incubated until reaching confluence. OGD/R modeling was then conducted, followed by the addition of 1 mg/mL FITC-dextran solution to the upper chamber. After 1 h of incubation, fluorescence intensity was measured using a fluorescence microplate reader, with excitation at 495 nm and emission at 520 nm (Xie et al., 2023).

### 2.14 Measurement of ROS levels

Third-generation cells were seeded at a concentration of 1 × 10^5^ cells per well in six-well plates. Once 70%–80% confluency was achieved, cells were exposed to OGD/R intervention. Intracellular ROS levels were quantified using a commercially available ROS detection kit (Elabscience, Wuhan, China) as per the instructions provided by the manufacturer. Fluorescence imaging was carried out with the help of a Nikon ECLIPSE CI-L microscope, and the acquired images were analyzed for fluorescence intensity using ImageJ software.

### 2.15 Statistical analysis

Statistical analyses were performed using IBM SPSS Statistics software (v26.0, IBM, USA). BBB scores was analyzed using repeated-measures ANOVA (RM-ANOVA), with simple effects tests conducted when significant interactions emerged. Between-group comparisons utilized one-way ANOVA, followed by LSD *post hoc* tests with Bonferroni correction for multiple comparisons. For data violating normality or homogeneity of variance assumptions, Kruskal–Wallis non-parametric tests were employed with appropriate *post hoc* corrections. Statistical significance was defined as a p-value below 0.05. GraphPad Prism 8.0 software was utilized for visualizing and plotting the data.

## 3 Results

### 3.1 IRFV administration enhances locomotor performance and reduces tissue pathology in rodent models of spinal cord trauma

**FIGURE 1:**
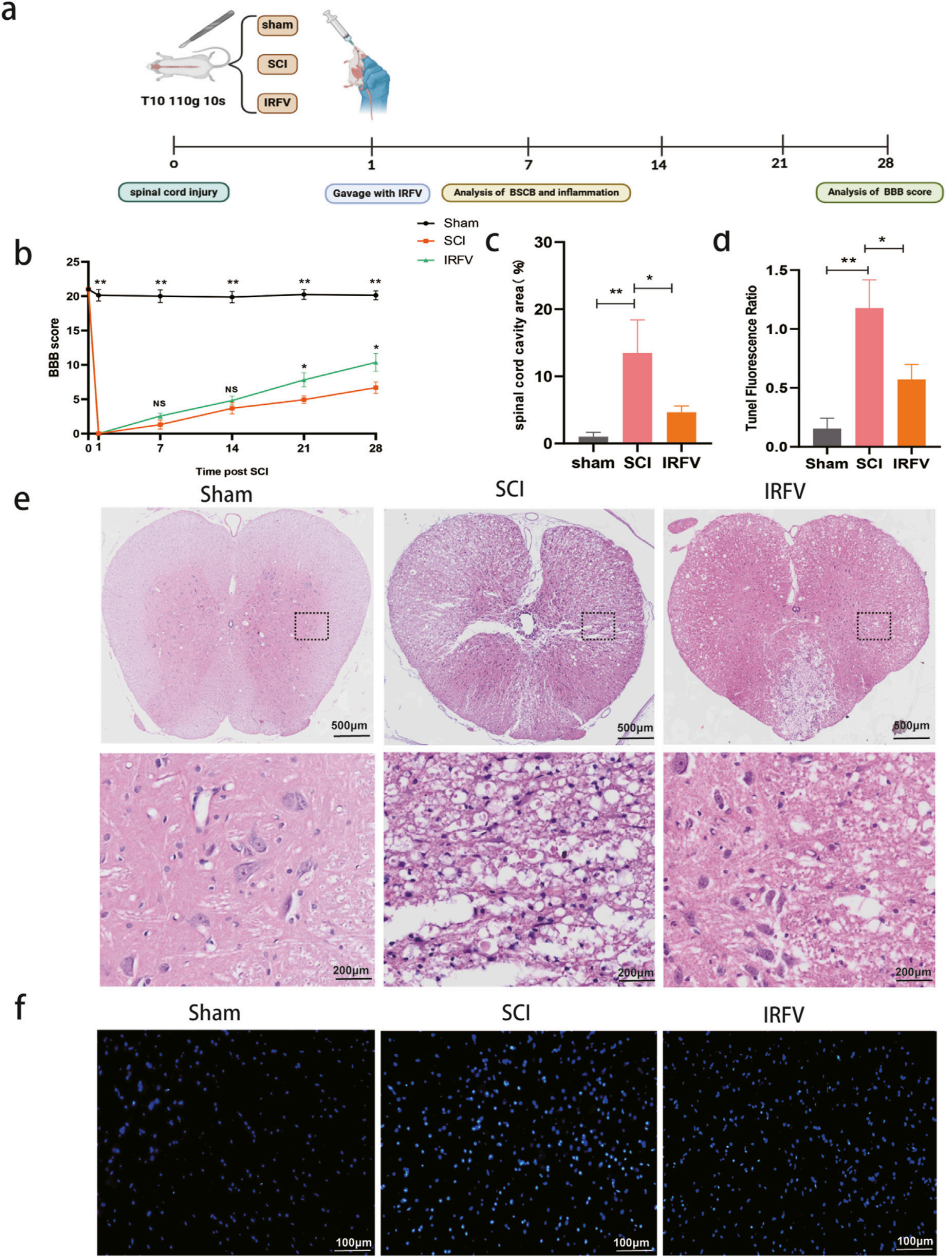
IRFV administration enhances locomotor performance and reduces tissue pathology in rodent models of spinal cord trauma. **(a)** Flow chart; **(b)** Basso-Beattie-Bresnahan score (*n* = 8); **(c)** Proportion of cavity area of spinal cord; **(d)** TUNEL staining fluorescence ratio; **(e)** H&E staining (*n* = 4); **(f)** TUNEL staining (*n* = 4). Statistical analysis revealed significant differences with SCI group (NS denotes non-significant result,*p < 0.05, **p < 0.01).

To evaluate IRFV-mediated functional recovery after spinal cord injury, locomotor performance was assessed via the BBB scale at pre-injury baseline and post-trauma intervals (days 1, 7, 14, 21, and 28). At 24 h post-injury, complete hindlimb paralysis (BBB score = 0) was observed in all injured cohorts, while sham-operated controls maintained normal function, confirming successful model induction. Longitudinal analysis revealed progressive locomotor improvement across injury groups. Critically, IRFV-treated animals exhibited statistically superior BBB scores at days 21 and 28 compared to vehicle-treated SCI controls (*p* < 0.05), demonstrating therapeutic efficacy in functional restoration (Figure 1b).

Pathological alterations post-SCI were analyzed using hematoxylin-eosin (H&E) staining. At 7 days post-injury, sham-operated controls maintained intact spinal cord architecture without significant damage. Conversely, SCI controls exhibited extensive tissue pathology featuring inflammatory cell infiltration, loss of gray-white matter demarcation, and large cavitation areas. IRFV treatment significantly attenuated these pathological changes (Figures 1c,e), demonstrating reduced inflammatory infiltration, preserved tissue boundaries, and smaller lesion cavities. To assess IRFV’s anti-apoptotic effects, TUNEL staining was performed (Figures 1d,f). Quantitative analysis revealed significantly reduced apoptotic cell density in IRFV-treated animals compared to vehicle-treated SCI controls (*p* < 0.05). These findings demonstrate that IRFV confers neuroprotection through suppression of apoptosis, corresponding with improved functional recovery and reduced histopathology in SCI models.

### 3.2 IRFV inhibited inflammatory response and immune infiltration after SCI

**FIGURE 2:**
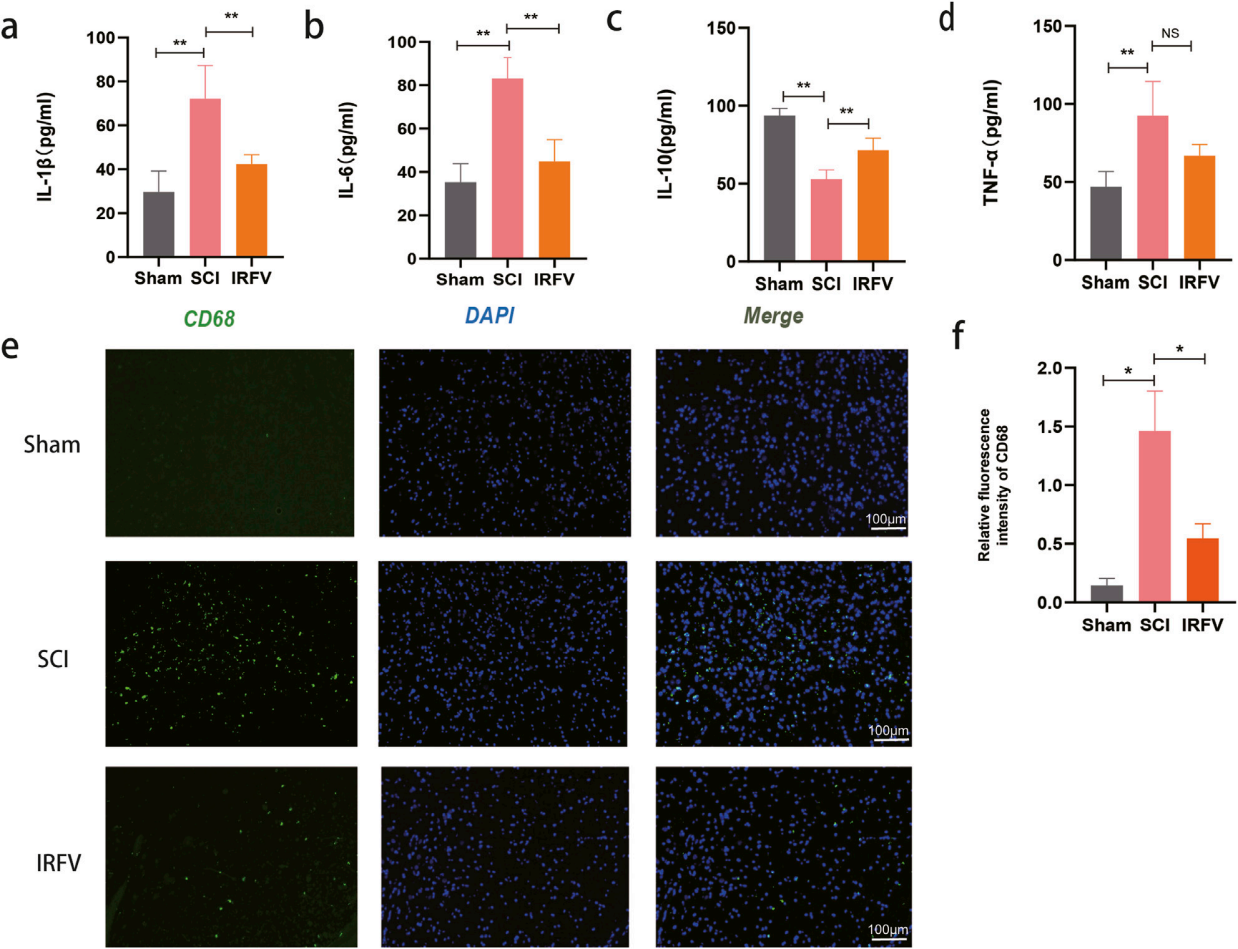
IRFV inhibited inflammatory response and immune infiltration after SCI. **(a–d)** Levels of the inflammatory cytokines IL-1β, IL-6, TNF-α, and the anti-inflammatory cytokine IL-10; **(e,f)** Immunofluorescence staining of the macrophage marker protein CD68, scale bar = 100 μm. *n* = 4. Statistical analysis revealed significant differences with SCI group (NS denotes non-significant results,*p < 0.05, **p < 0.01).

The disruption of BSCB integrity post-spinal cord trauma leads to vascular leakage, immune cell migration, and increased inflammatory mediator release, ultimately driving neuroinflammatory cascades in SCI. Neuroinflammation serves as a pivotal mediator of secondary pathogenesis following spinal cord trauma, substantially impeding endogenous repair mechanisms within the injured neural tissue. This study investigated the effects of IRFV intervention on neuroinflammation. The concentrations of inflammatory mediators, including pro-inflammatory cytokines (IL-1β, IL-6, TNF-α) and anti-inflammatory cytokine IL-10, were measured in spinal cord homogenates through ELISA analysis. IRFV treatment significantly reduced the levels of IL-6, IL-1β, and TNF-α, (*p* < 0.01) while simultaneously increasing IL-10 expression, compared to the SCI group (Figures 2a–d).

To assess immune cell infiltration in spinal cord tissue, immunofluorescence analysis was performed using CD68 (a macrophage marker) (Figures 2e,f). Results revealed a significant increase in macrophage infiltration following SCI. However, IRFV intervention significantly attenuated the macrophage infiltration (*p* < 0.05). These findings support the potential of IRFV to suppress the inflammatory response and reduce immune cell infiltration following SCI, which may contribute to mitigating secondary injury and promoting tissue repair.

### 3.3 IRFV alleviates BSCB permeability and ultrastructure damage after SCI

**FIGURE 3:**
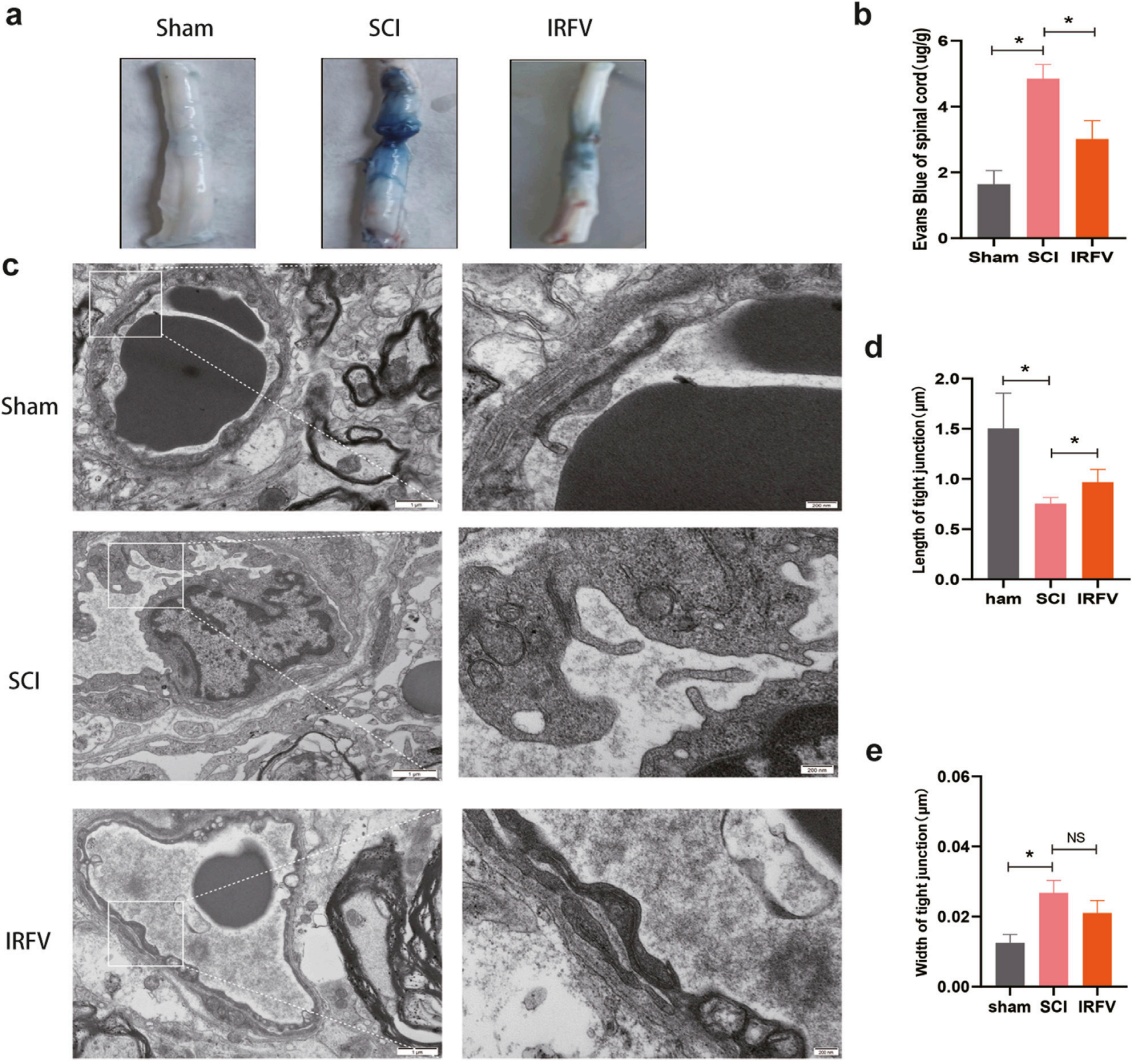
IRFV alleviates BSCB permeability and ultrastructure damage after SCI. **(a)** Spinal cord tissue was analyzed by Evans blue staining (*n* = 3); **(b)** Evans blue content in spinal cord tissue; **(c)** Transmission electron microscopy analysis of tight junctions (*n* = 4). **(d,e)** Analysis of tight junction length and width.; scale bar = 1 μm (left), 200 nm (right). Statistical analysis revealed significant differences with SCI group (NS denotes non-significant results,*p < 0.05, **p < 0.01).

The BSCB plays a key role in restricting inflammatory responses by regulating permeability. Evans blue dye, which cannot traverse the intact BSCB under physiological conditions, is commonly used to assess barrier integrity. Increased BSCB permeability permits extravasation of macromolecules such as Evans blue, thereby providing a direct metric for barrier dysfunction. Compared to the Sham group, Evans blue content was significantly elevated in the spinal cord tissue of the SCI group (*p* < 0.05), confirming increased BSCB permeability. IRFV intervention markedly reduced Evans blue extravasation relative to the SCI group (Figures 3a,b). These data demonstrate that IRFV ameliorates BSCB hyperpermeability following spinal cord injury.

The functional integrity of the BSCB relies on its structural integrity. We analyzed post-SCI structural alterations in the BSCB using transmission electron microscopy (TEM) (Figures 3c–e). TEM revealed significant structural impairment in the SCI group, characterized by: disruption of the basement membrane, altered endothelial cell morphology, and disrupted tight junctions. IRFV intervention ameliorated these structural defects, increasing basement membrane thickness and tight junction length compared to the SCI group. These results demonstrate that IRFV intervention protects BSCB structural integrity following SCI.

### 3.4 IRFV lessened the degradation of tight junction proteins in the BSCB

**FIGURE 4:**
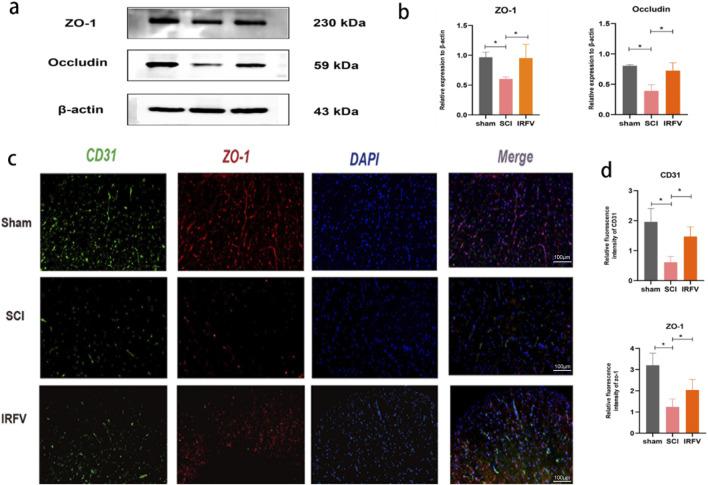
IRFV lessened the degradation of tight junction proteins in the BSCB. **(a,b)** The analysis of ZO-1 and Occludin protein expression in spinal cord tissue via Western blot (*n* = 3); **(c,d)** The expression of CD31 and ZO-1 in spinal cord sections was examined by means of immunofluorescence analysis. (*n* = 4), scale bar = 100 μm. Statistical analysis revealed significant differences with SCI group (NS denotes non-significant results,*p < 0.05, **p < 0.01).

Tight junctions play a pivotal role in modulating the permeability of the BSCB. To elucidate this mechanism, we analyzed the expression of tight junction proteins in the BSCB. Western blot analysis revealed that IRFV treatment significantly upregulated the expression of ZO-1 and Occludin compared to the SCI group, suggesting that IRFV promotes the restoration and maintenance of BSCB function by enhancing the expression of these key proteins, thereby regulating barrier permeability (Figures 4a,b). This finding was further corroborated by immunofluorescence microscopy (Figures 4c,d). We first labeled vascular endothelial cells with the marker CD31 and observed reduced CD31 expression after SCI, indicating vascular damage. Through immunofluorescence colocalization, we found that IRFV treatment notably increased the expression of the tight junction protein ZO-1, as evidenced by intensified fluorescence signals at the endothelial cell junctions. Collectively, these results demonstrate that IRFV intervention prevents the downregulation of tight junction proteins in the BSCB, which may represent a key mechanism by which IRFV modulates BSCB permeability.

### 3.5 IRFV may play a role in regulating Nrf2/HO-1 pathway

**FIGURE 5:**
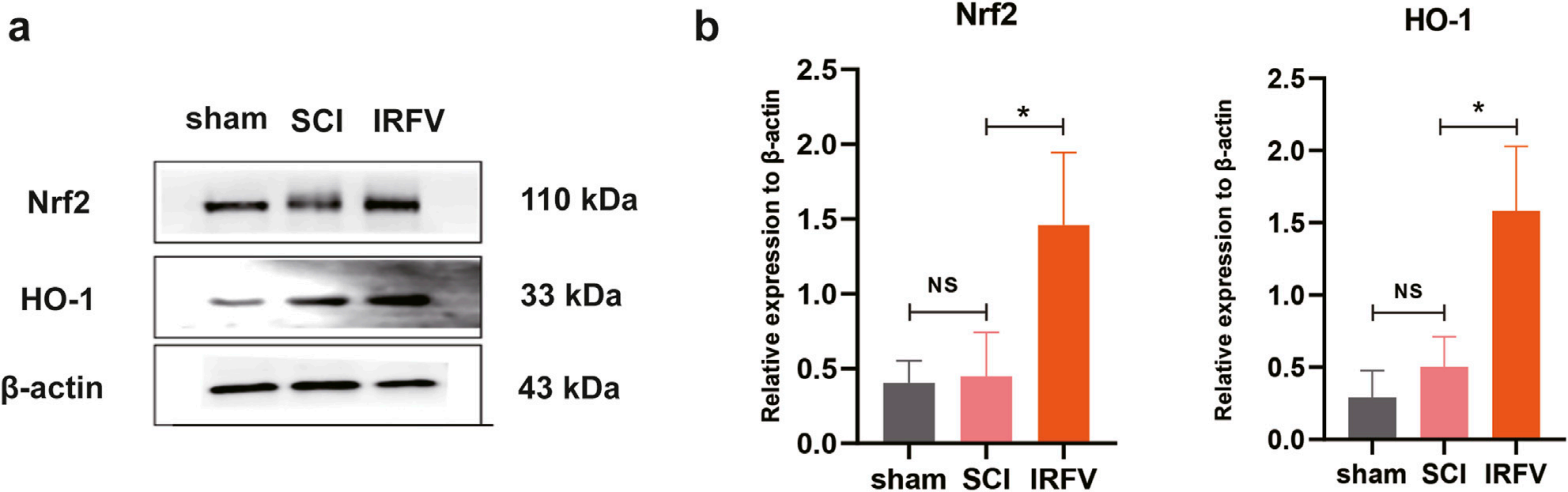
IRFV may play a role in regulating the Nrf2/HO-1 pathway **(a,b)**. Western blot analysis of protein Nrf2 and HO-1 expression (*n* = 3). Statistical analysis revealed significant differences with SCI group (NS denotes non-significant results,*p < 0.05, **p < 0.01).

The Nrf2/HO-1 signaling pathway is a well-characterized mechanism governing oxidative stress homeostasis and inflammatory responses. Activation of Nrf2 nuclear translocation potently upregulates HO-1 protein expression, with HO-1 playing a critical role in safeguarding the BSCB by mitigating its damage through multiple protective mechanisms. Using Western blot analysis, we quantified the protein expression of key components in the Nrf2/HO-1 pathway within spinal cord tissues. Results demonstrated that IRFV intervention significantly enhanced the expression of both Nrf2 and HO-1 proteins compared to the SCI group (*p* < 0.05). These findings suggest that IRFV may ameliorate BSCB damage and facilitate spinal cord injury recovery by activating the Nrf2/HO-1 signaling axis (Figure 5).

### 3.6 IRFV improves the viability of HCMEC/D3 cells and reduces apoptosis caused by OGD/R-induced injury

**FIGURE 6:**
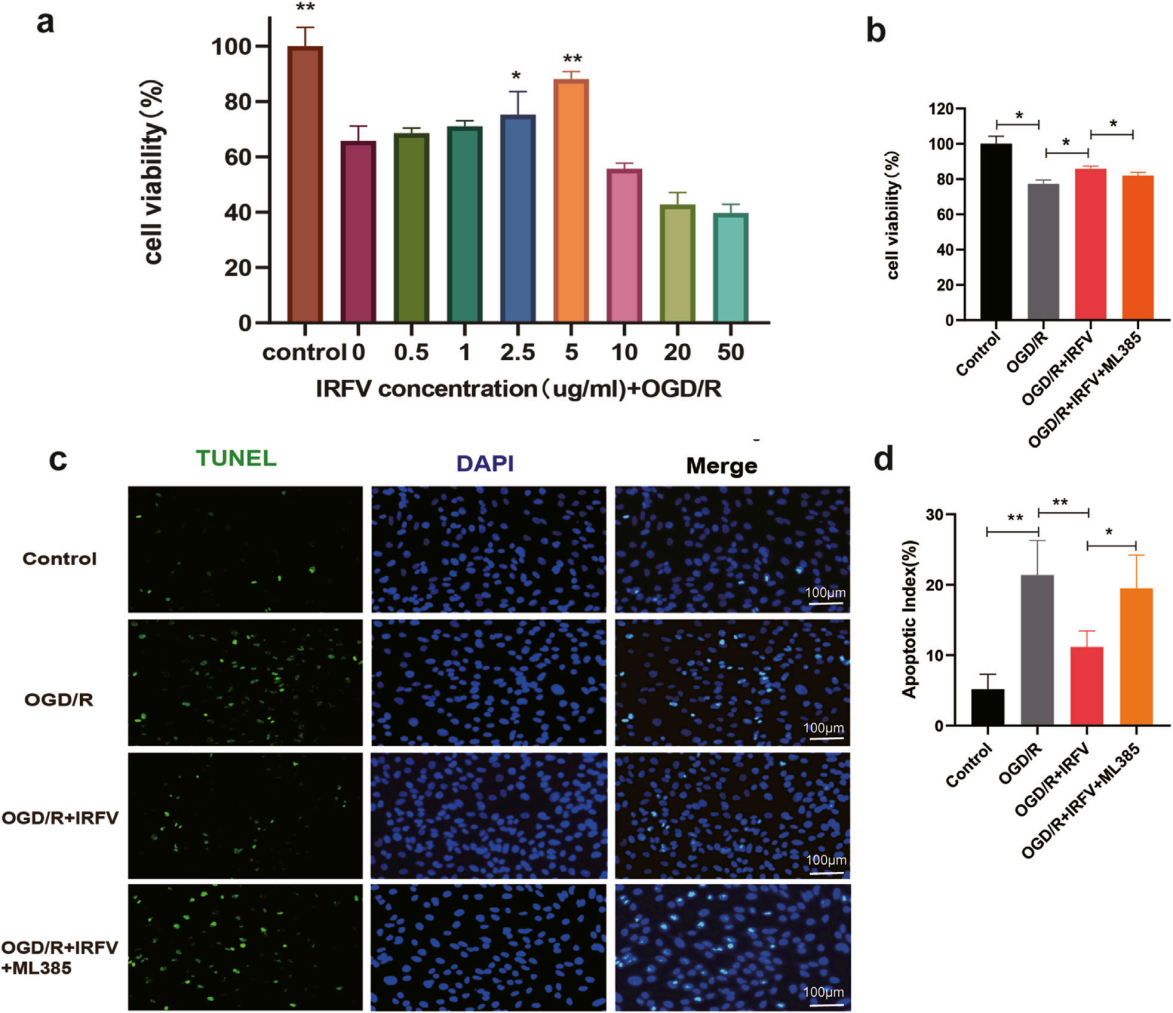
IRFV improves the viability of HCMEC/D3 cells and reduces apoptosis caused by OGD/R-induced injury. **(a)** Cell viability under different concentrations of IRFV intervention; **(b)** Viability of HCMEC/D3 cells in each group; **(c)** Percentage of apoptotic-positive HCMEC/D3 cells in each group. *n* = 4, scale bar = 100 μm; **(d)** Apoptosis status of HCMEC/D3 cells in each group; Statistical analysis revealed significant differences with OGD/R group, with OGD/R + IRFV (*p < 0.05, **p < 0.01).

To further elucidate the protective mechanisms of IRFV on the BSCB, an *in vitro* oxygen-glucose deprivation/reoxygenation (OGD/R) model was established using hCMEC/D3 cells to mimic BSCB disruption. The results showed that a 5 μg/mL IRFV solution significantly ameliorated cell viability (*p* < 0.01), thus establishing this concentration as the optimal dosage for subsequent assays (Figure 6a). Compared with the OGD/R group, IRFV intervention markedly enhanced cell viability (*p* < 0.05); however, co-treatment with the Nrf2 inhibitor ML385 abrogated this protective effect, underscoring the essential role of the Nrf2 signaling pathway (Figure 6b). TUNEL staining further confirmed that IRFV intervention suppressed endothelial cell apoptosis induced by OGD/R (*p* < 0.01), whereas ML385 cotreatment reversed this effect, leading to a significant increase in apoptotic cells (Figures 6c,d). Collectively, these findings demonstrate that IRFV effectively mitigates OGD/R-induced endothelial cell death, with the Nrf2 signaling pathway playing a pivotal role in mediating this protective response.

### 3.7 IRFV reduces the degradation of tight junction proteins by alleviating oxidative stress

**FIGURE 7:**
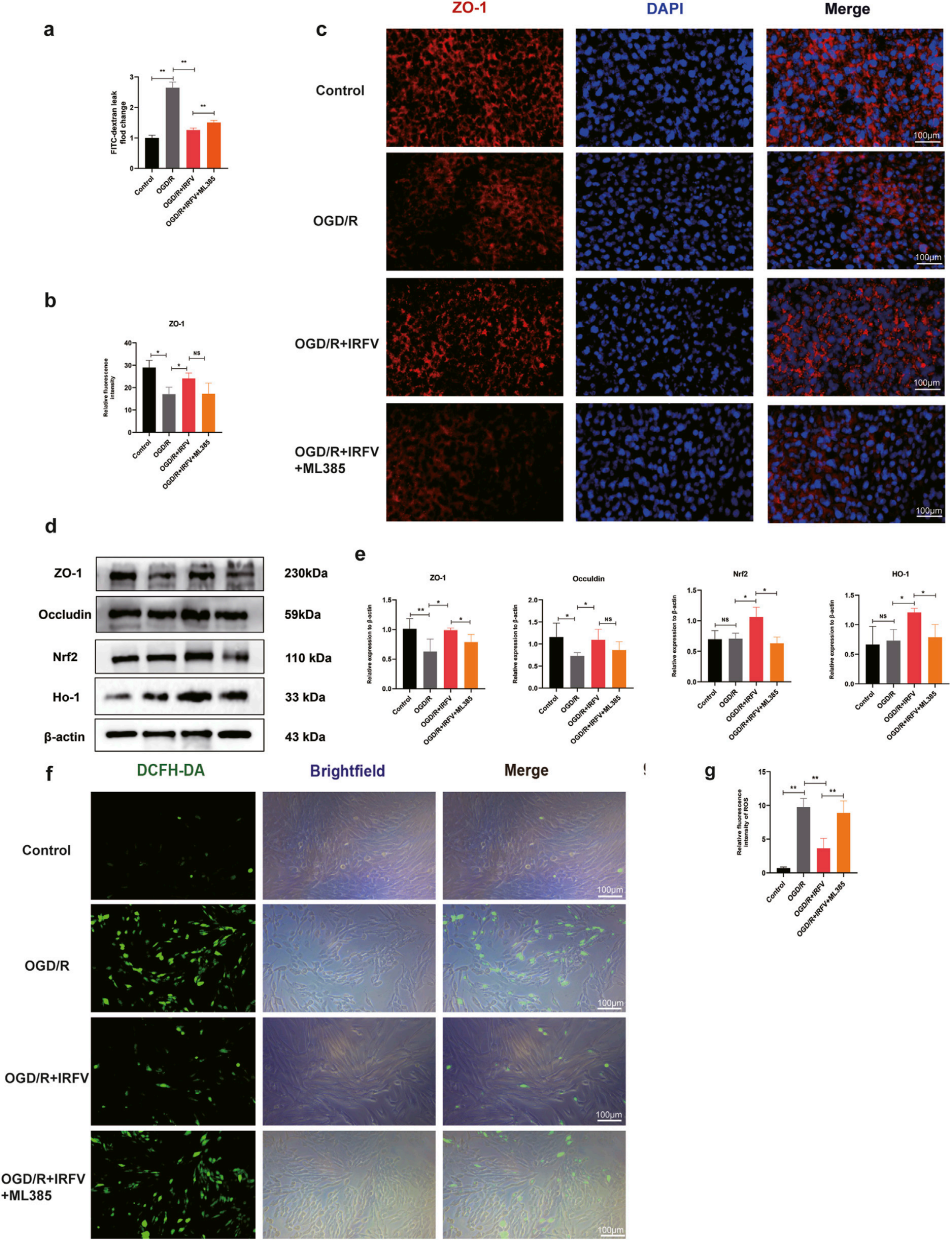
IRFV reduces the degradation of tight junction proteins by alleviating oxidative stress. **(a)** Barrier permeability assay; **(b,c)** Immunofluorescence analysis of ZO-1 tight junction protein expression, scale bar = 100 μm. **(d,e)** The expression of Nrf2, HO-1, ZO-1, and Occludin proteins was analyzed using Western blot; **(f,g)** ROS content measurement. *n* = 4, scale bar = 100 μm. Statistical analysis revealed significant differences with OGD/R group, with OGD/R + IRFV (NS denotes non-significant results,*p < 0.05, **p < 0.01).

We further investigated the disruption of tight junction. The results demonstrated that the OGD/R group exhibited a significant increase in fluorescence intensity compared to the control group, indicating enhanced cell permeability and compromised tight junction integrity. Conversely, IRFV treatment notably decreased permeability and partially restored barrier function relative to the OGD/R group (*p* < 0.05). However, the addition of the Nrf2 inhibitor ML385 abrogated this beneficial effect of IRFV, as evidenced by a discernible increase in permeability (Figure 7a). Immunofluorescence and Western blot analyses further revealed that IRFV intervention significantly upregulated the expression of the tight junction proteins ZO-1 and Occludin compared to the OGD/R group (*p* < 0.05). ML385 co-treatment abolished IRFV-induced upregulation of tight junction proteins (ZO-1/Occludin) in the OGD/R + IRFV + ML385 group (Figures 7b–e). These findings collectively suggest that IRFV mitigates tight junction disruption, and the Nrf2 signaling pathway is essential for mediating this protective effect.

Oxidative stress has a vital part in tight junction disruption. ROS fluorescence assays indicated that the ROS levels in the OGD/R group were notably higher than those in the control group. In comparison with the OGD/R group, IRFV treatment remarkably decreased the levels of reactive oxygen species (*p* < 0.01). However, the addition of ML385 weakened the antioxidant properties of IRFV (Figures 7f,g). Through Western blot analysis of the Nrf2/HO-1 signaling pathway, it was found that IRFV intervention significantly increased the expression of Nrf2 and HO-1 proteins as opposed to the OGD/R group. However, ML385 suppressed the promotive effects of IRFV on Nrf2 and HO-1 expression (Figures 7d,e). In conclusion, IRFV improves the viability of endothelial cells, decreases apoptosis, stimulates the expression of tight-junction proteins ZO-1 and Occludin. When the Nrf2 inhibitor ML385 was added, the positive effects of IRFV were nullified. This suggests that the therapeutic effects of IRFV are achieved through the activation of the Nrf2/HO-1 signaling pathway.

## 4 Discussion

Spinal cord injury (SCI) represents a devastating medical condition, often leading to irreversible disability and profound functional impairment (Anjum et al., 2020; French et al., 2007). The global annual incidence of SCI is estimated to range between 250,000 and 500,000 cases (Khorasanizadeh et al., 2019). Despite extensive research efforts dedicated to unraveling the complex pathophysiology of SCI and developing targeted therapies, clinical translation remains challenging. Currently, methylprednisolone is the only FDA-approved pharmacological treatment for SCI, yet its severe adverse effects substantially limit clinical applicability, underscoring the urgent need for novel therapeutic agents (Karsy and Hawryluk, 2019). IRFV, an iridoid compound extracted from VJJ, has demonstrated promising therapeutic potential in neurological disorders, including anxiolytic (Zhang et al., 2018), antidepressant (Li et al., 2020), and anti-post-traumatic stress disorder activities (Yang et al., 2021). Previous studies from our laboratory have shown that IRFV promotes axonal regeneration during the chronic phase of SCI (Wang et al., 2024) and enhances hindlimb motor function recovery. However, the precise mechanisms underlying its therapeutic efficacy remain unclear. To address this knowledge gap, we conducted a series of experiments aimed at elucidating the potential therapeutic effects and underlying mechanisms of IRFV in SCI.

Our study demonstrated that IRFV intervention significantly enhanced motor function recovery in SCI rats at 21 and 28 days post-injury compared to the SCI group. On day 7 post-SCI, IRFV mitigated histopathological damage and reduced apoptosis in spinal cord tissue, suggesting a potential mechanism for its later functional benefits. However, at earlier timepoints (days 7 and 14), IRFV only modestly increased BBB scores without achieving statistical significance versus the SCI group. This delayed functional recovery likely reflects the severity of neural circuitry disruption in acute SCI. Neural network restoration and reorganization typically occur during later stages of spinal cord injury (Anjum et al., 2020). Thus, while IRFV attenuated acute spinal cord pathology, significant motor functional recovery became evident only during the subacute and chronic phase.

Neuroinflammatory responses represent a critical component of secondary injury following SCI (Anjum et al., 2020), disrupting the local microenvironment, suppressing neurotrophic factor secretion, and promoting glial scar formation—all of which impede neural regeneration (Liu et al., 2023). Reducing inflammation has been shown to facilitate motor function recovery (Xuan et al., 2023), prompting us to investigate the anti-inflammatory effects of IRFV. Macrophage infiltration into spinal cord tissue is a hallmark of the inflammatory response, with peak accumulation occurring on day 7 post-SCI (Tail et al., 2024). Thus, we chose day 7 as the time point to analyze inflammatory changes. Our findings show that IRFV intervention significantly suppresses the secretion of inflammatory factors, alleviates apoptotic cell death in spinal cord tissues, and mitigates pathological damage on day 7 after SCI. During the early phase of SCI, macrophages progressively infiltrate the spinal cord, releasing pro-inflammatory cytokines that recruit neutrophils and microglia, thereby exacerbating neuroinflammation (Kwiecien et al., 2020; Ahuja et al., 2017). Fan et al. (2022) demonstrated that hydrogels modulate neuroinflammation to promote axonal regeneration and functional recovery after SCI. Consistently, our study reveals that IRFV restricts macrophage infiltration and dampens inflammatory responses. These results suggest that during the early post-injury period, IRFV exerts its therapeutic effects by regulating the immune response, limiting inflammatory cell infiltration, and inhibiting neuroinflammatory cascades.

Following spinal cord injury, alterations in BSCB permeability represent a critical node in the progression of inflammatory responses. Studies have shown that preserving BSCB integrity can mitigate inflammatory cascades, reduce neuronal loss, and promote functional recovery after spinal cord injury (Nie et al., 2024). Our findings suggest that IRFV intervention decreases BSCB permeability, which may serve as a specific mechanism by which IRFV alleviates neuroinflammation. BSCB permeability is inherently dependent on the structural integrity of its components (Tail et al., 2024). Using transmission electron microscopy, we observed structural changes in the BSCB. In the SCI group, the BSCB basement membrane exhibited thinning, endothelial cells displayed morphological abnormalities, and tight junctions were disrupted. By contrast, IRFV intervention reduced tight junction gaps, increased junctional length, and promoted basement membrane thickening. These structural improvements likely underlie the mechanism by which IRFV enhances BSCB permeability regulation.

Tight junctions serve as the functional units of the blood-spinal cord barrier (BSCB), restricting the paracellular entry of external substances to maintain neural microenvironmental homeostasis; disruption of these junctions leads to BSCB dysfunction (Jin et al., 2021; Wang Q. et al., 2023). Further investigations revealed that IRFV intervention upregulates the expression of tight junction proteins ZO-1 and Occludin, which serve as key regulators of junctional integrity and reflect the functional status of BSCB structures (Górecka et al., 2024; Jin et al., 2021). We hypothesize that IRFV mitigates BSCB disruption by modulating tight junction protein expression and organization. Cellular experiments demonstrated that OGD/R-injured endothelial cells exhibited reduced tight junction protein expression and increased intercellular permeability. Conversely, IRFV treatment promoted tight junction protein expression and restored barrier permeability. These results confirm that IRFV reduces BSCB permeability by attenuating tight junction protein degradation, thereby preserving barrier function.

The activation of Nrf2 by IRFV likely involves multiple interconnected mechanisms. Studies have shown that IRFV and its active components directly modulate reactive oxygen species (ROS) levels during oxidative stress (Jain et al., 2018). Oxidative stress disrupts the Keap1-Nrf2 interaction, promoting Nrf2 nuclear translocation and subsequent transcriptional activation (Ulasov et al., 2022). I Thus, IRFV may modulate the Nrf2 signaling axis by regulating oxidative stress homeostasis. Moreover, our previous study demonstrated that IRFV activates the PI3K/Akt pathway, leading to enhanced Akt phosphorylation (Wang et al., 2024). Phosphorylated Akt facilitates Nrf2 nuclear translocation, playing a crucial role in oxidative stress resistance and inflammatory regulation (Zhang et al., 2023). This suggests an indirect mechanism by which IRFV regulates Nrf2, potentially through PI3K/Akt-mediated signaling crosstalk.

The Nrf2/HO-1 pathway, a classical anti-inflammatory and antioxidative signaling pathway, plays a critical role in multiple diseases, such as SCI (Guo et al., 2023), ischemic brain injury (Chumboatong et al., 2020), and rheumatoid arthritis (Tang et al., 2024). Under basal conditions, Nrf2 is retained in the cytoplasm by Keap1 and marked for proteasomal degradation. However, under conditions of oxidative or electrophilic stress, this interaction is disrupted, enabling Nrf2 to translocate to the nucleus where it activates antioxidant response element (ARE)-driven genes such as HO-1, NQO1, and various glutathione-related enzymes. This mechanism plays an essential role in mitigating oxidative damage, as evidenced by studies involving liver injury and ischemia-reperfusion models in which Nrf2 activation significantly reduces cellular harm. In addition to its role in antioxidant defense, Nrf2 exerts context-dependent effects on inflammation. It generally suppresses excessive inflammation by inhibiting NF-κB signaling pathways and pro-inflammatory cytokines (e.g., TNF-α and IL-6), as demonstrated in lung injury models where deficiency of Nrf2 exacerbates tissue damage. Conversely, Nrf2 can also promote certain inflammatory mediators (e.g., IFN-γ and TGF-β) that facilitate tissue repair, underscoring its involvement in balancing immune responses. This dual functionality renders Nrf2 a promising yet complex therapeutic target; while its activation may confer protection against oxidative stress-related diseases and inflammatory disorders, sustained overactivation could potentially contribute to pathological conditions such as cancer. Xuan et al. (2023) demonstrated that PHR facilitates motor function recovery after SCI by modulating the Nrf2/HO-1 pathway. Animal experiment results showed that IRFV reduced inflammation and blood-spinal cord barrier permeability while promoting the expression of Nrf2/HO-1 pathway proteins. We hypothesize that IRFV exerts its effects by activating the Nrf2/HO-1 pathway. Furthermore, IRFV treatment activated Nrf2 and HO-1 protein expression, and suppression of Nrf2 expression abrogated IRFV’s protective effect on tight junctions. Taken together, IRFV may reduce inflammatory response and BBB permeability by activating Nrf2/HO-1 signaling pathway.

Finally, this study has some limitations due to time and experimental techniques. In order to observe the peak inflammatory response, we set the study observation time as 7 days after surgery. Therefore, the changes of inflammatory response and BSCB permeability at different time points could not be observed. In addition, we only observed the changes of BSCB and inflammatory response, and failed to analyze the interaction between these two factors. In the future, we will conduct a comprehensive analysis of inflammation and BSCB changes at different time points following spinal cord injury, and further investigate the interaction between blood-spinal cord barrier disruption and the inflammatory response. Pharmacokinetic (PK) studies on IRFV remain sparse in the literature, with no consensus on its absorption and elimination profiles. For instance, Sun et al. (2015) demonstrated in a study using six male Wistar rats that valtrate—a bioactive iridoid compound—exhibited extensive distribution (volume of distribution: 49.56 L/kg) and rapid systemic elimination (clearance rate: 31.43 L/(hkg); half-life: 1.12 h). In future studies, we plan to develop advanced delivery systems (e.g., hydrogels, nanomaterials) to optimize spinal cord-targeted delivery of IRFV, thereby enhancing its therapeutic efficacy and bioavailability. Concurrently, we will characterize IRFV’s pharmacokinetic profile and identify its active components responsible for observed therapeutic effects. These investigations will further elucidate Valeriana jatamansi Jones’ pharmacological mechanisms and accelerate its clinical translation for spinal cord injury treatment.

## 5 Conclusion

Our study demonstrates that IRFV ameliorates neuroinflammation following SCI by reducing macrophage infiltration and pro-inflammatory cytokine secretion. Concurrently, IRFV restores BSCB integrity by mitigating structural damage and preventing tight junction protein degradation. Mechanistically, IRFV likely attenuates immune cell infiltration and inflammatory mediator entry into the spinal cord parenchyma through its BSCB-protective effects. Critically, we propose that IRFV exerts these beneficial actions via activation of the Nrf2/HO-1 signaling pathway. Collectively, this work highlights the therapeutic potential of IRFV for SCI treatment and provides a mechanistic foundation for its future clinical translation and drug development.

## Data Availability

The original contributions presented in the study are included in the article/Supplementary Material, further inquiries can be directed to the corresponding author.
