# Supplementary figures and images for: Iridoids derived from Valeriana jatamansi Jones alleviates neuroinflammation and blood spinal cord barrier permeability after spinal cord injury by activating the Nrf2/HO-1 signaling pathway
Source: Front Pharmacol. 2025 Jul 18;16:1597719. doi: 10.3389/fphar.2025.1597719 (PMC12313594; doi:10.3389/fphar.2025.1597719)

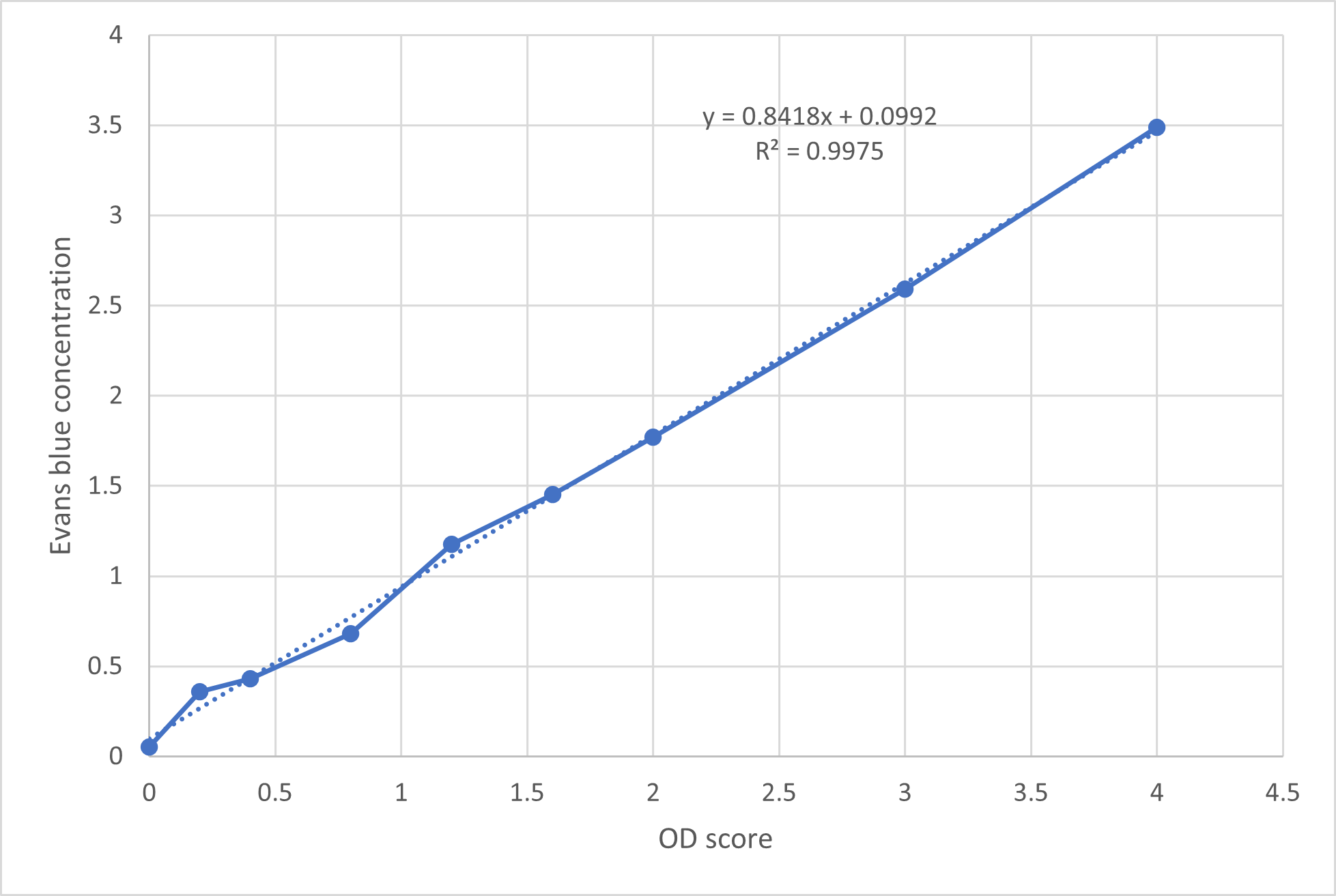

Supplement: Supplementary file 1 [file Image1.tif]
